# Resveratrol regulates body weight in healthy and ovariectomized rats

**DOI:** 10.1186/s12986-017-0183-5

**Published:** 2017-04-14

**Authors:** Rupali Sharma, Neel Kamal Sharma, M. Thungapathra

**Affiliations:** 1 Department of Biochemistry, PGIMER, Chandigarh, India; 2 Armed Forces Radiobiology Research Institute, USUHS, 8901 Wisconsin Avenue, Bethesda, MD USA

**Keywords:** Ovariectomy, Resveratrol, OVX, Soya free diet

## Abstract

**Objective:**

The elevated body weight in post-menopausal state attributes to the reduced estrogen levels which is alleviated by resveratrol (RES) but its role in control rats is not well understood. The main objective of the study was to explore the effects of RES on the body weight of ovariectomized (OVX) female rats with controls and to relate their biochemical parameters.

**Methods:**

Female Wistar rats weighing 200–300 g underwent bilateral ovariectomy (OVX) and were fed soya free diet (*n =* 8 rats per group). In all groups: (Control, Control + Resveratrol, OVX and OVX + Resveratrol) resveratrol was administered orally at a dose of 5 mg/kg/day for 1 month. Glucose and other biochemical parameters were examined.

**Results:**

Significant reduction in the gain of body weight was observed in the control rats treated with resveratrol. Ovariectomy caused an escalation in gain of body weight due to loss of estrogen which was brought down with resveratrol. There was a slight dip in the blood glucose levels after resveratrol treatment.

**Conclusion:**

Resveratrol significantly reduced the gain of body weight in the control rats and in OVX rats showing its antiobesogenic effects.

## Background

For the prevention of menopausal symptoms estrogens has long been tried with alterations in the dosing and route of administration, but it has myriad of side effects, which includes the risk of endometrium and breast cancer [1, 2]. The need of the hour is to find out some alternatives so we thought of resveratrol which is an antioxidant and is popularly called as a fountain of youth.

Resveratrol (RES) is a phytoestrogen with all its estrogenic, antioxidant, anti-inflammatory properties found in a diverse plant species. It is popular for all its health benefits, especially in type 2 diabetes, cardiovascular disorders, cancer and neurological disorders.

Resveratrol is known to imitate energy restriction in high-fat diet fed rat and mice. RES effectively combated gain in body weight, decreased intra - abdominal fat, and dropped serum triglycerides, cholesterol, and free fatty acids (FFA) levels in obese Zucker fatty rats [3]. Little is known regarding the safety of RES in control rats and hardly any evidence exists concerning its effect on body weight. We therefore set out to study the effects of RES on body weight in the control and ovariectomized (OVX) rats. Recently, it was found that there is no adjuvant effect of RES on energy restriction for obesity treatment [4]. In accordance with the study of Majumdar et al. [5], we also found that ovariectomy resulted in an increase in the body weight and RES had the potential to bring down the gain in body weight due to ovariectomy. At present there are many therapeutic agents that decrease body weight but they have contrary side effects, not many therapeutic agents are known that decrease body weight without causing severe side effects. Though there has been a great deal of studies on the protective effects of resveratrol on obesity using rat as a model but there were reports on its effects on body weight especially in the control rats. We found that when resveratrol was given to control rats and OVX rats there was a reduction in their gain of body weight in comparison to their respective untreated groups. We want to address here that why resveratrol acts in a differential manner in the control and OVX (menopausal model) rats.

## Methods

### Animals and dose preparation

Female wistar rats weighing 200–300 g, were housed at the central animal house (PGIMER, Chandigarh, India) and were acclimatized for 1 week in cages (*n =* 1/cage) in a 12 – hr light and dark cycle and a constant temperature of 25 – 27 ^ο^C. Standard soya free, isonitrogenous and isocaloric diet was made available ad libitum with free access to drinking water. PGIMER ethical clearance was taken (PGIMER 60/IAEC/329) and all the animals were approved by animal ethics committee. Rats were handled and euthanized in full agreement with the institutional guidelines for the care and management of laboratory animals. Rats were randomly divided into four groups- (i) Normal rats as control group (ii) Normal rats with resveratrol (Receiving resveratrol treatment for 1 month) (iii) Ovariectomized (OVX) rats (iv) OVX rats with resveratrol (One month of resveratrol treatment after 45 days of OVX).

Animals requiring resveratrol treatment were fed with a diet containing 5 mg/kg body weight/day of resveratrol for one month. For this, resveratrol (SigmaAldrich, USA) was dissolved in 70% ethanol and added to the ground diet to make the homogenous dough. The dough was then converted to pellet form and kept for drying at room temperature for 1 day. The diet pellets containing resveratrol were stored at - 20 ° C for up to one week. Animals in the resveratrol treatment groups were first given resveratrol containing pellets (after calculating the required weight of the pellets based on the individual animal body weight) and once they have been finished, the diet tray was replenished with diet without resveratrol. Dose preparation and administration were made in dark to evade isomerization of trans-resveratrol to the cis form. After the treatment period, the rats were kept for fasting overnight and were sacrificed. Blood was withdrawn by cardiac puncture for estimation of serum parameters.

### Bilateral ovariectomization

Prior to surgery each rat was anesthetized with an intraperitonial injection of ketamine hydrochloride (50 mg/kg) and xylazine (5-10 mg/kg). The success of anesthesia was evaluated by failure of the wink reflex and by the lack of reaction to pinching of the foot. Using aseptic conditions, a skin incision was made at the dorsal midline slightly caudal to the last rib followed by a flank incision of the abdominal muscles. The ovary was exteriorized. The proximal end of the uterine horn was then ligated with absorbable sutures (4–0 chronic gut), incised and removed with the attached ovary. The second ovary was removed from the opposite side in the same manner. Using absorbable sutures, the abdominal muscle incision was closed. Betadine was applied on the stitches after the surgery and were placed on heating pad (low setting), and covered with a surgical drape to minimize loss of body heat. Rats were returned to their cages only after complete recovery from anesthesia. Confirmation of ovariectomization was done by histology of ovary.

### Body weight and uterine weight

Body weight of animals was measured weekly. Weight gains were calculated by subtracting initial body weight with the final body weight immediately before sacrifice. At the time of sacrifice, uteri were carefully removed and cleaned of fat tissue. The actual uterus weight in grams was divided by the actual body weight of the animal and multiplied by the average body weight of animals of that group. Thus, the adjusted uterus weight was calculated by the given formula:

$$ \begin{array}{l}\mathrm{Adjusted}\ \mathrm{uterus}\ \mathrm{weight}=\frac{\mathrm{Weight}\ \mathrm{of}\ \mathrm{uterus}}{\mathrm{Weight}\ \mathrm{of}\ \mathrm{animal}} \times \mathrm{Average}\ \mathrm{weight}\ \mathrm{of}\ \mathrm{animal}\\ {}\end{array} $$

### Clinical chemistry

Blood from tail clipping was collected once after one week of lab acclimatization of the animal, and once at the time of euthanization. Blood glucose was estimated using glucometer (Accurex) following the manufacturer’s instructions. At the time of sacrifice, blood was collected by cardiac puncture for estimating the biochemical parameters such as urea, creatinine, triglycerides, cholesterol, high-density lipoprotein (HDL) and low-density lipoprotein (LDL), which were determined with a kit (Roche/Hitachi P - 800 clinical chemistry analyzer (Mannheim, Germany) with reagents from Roche Diagnostics.

###  Statistics

Data analysis was carried out using SPSS statistics software version 16.0 (Statistical Package for the Social Sciences) Statistical analysis was done. To check the normality of the data normal-Quantile (Q-Q) plots were constructed. After establishing the normality, unpaired student t- test, and one- way ANOVA (Analysis of variance) was applied. A *p* -value <0.05(*) and *p-*value <0.005(**) were considered statistically significant.

## Results

**Fig. 1:**
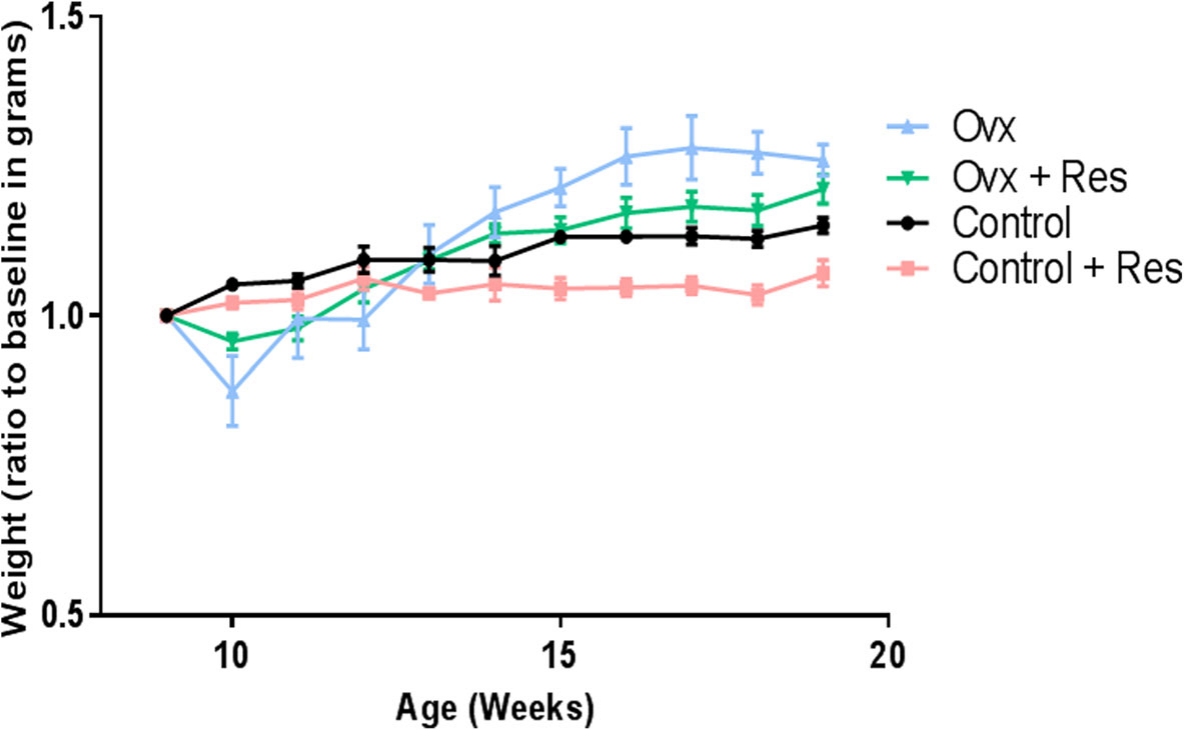
Body weight ratio to base line for different groups, ovx: ovariectomized, res: resveratrol

Body weight ratio to base line for Ovx, Ovx + Res, control and control + Res group is given in Fig. 1.

###  Effect of resveratrol on gain in body weight

**Fig. 2:**
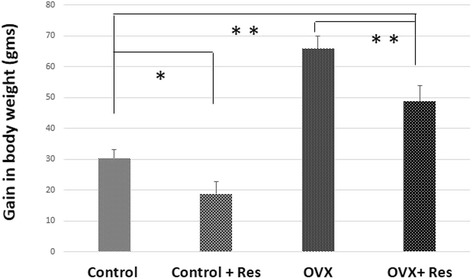
Effect of resveratrol on body weight in control, control + Res, OVX and OVX + Res rats

Significant weight loss occurred in the treatment groups (control treated and OVX treated) (*p <* 0.05) in comparison to control untreated animals. Due to loss of estrogen many of the metabolic pathways come to play role in ovariectomized group leading to significant gain in the body weight in comparison to the control untreated group (*p <* 0.05) (Fig. 2) which was significantly brought down by resveratrol.

### Effect of resveratrol on uterus weight

**Fig. 3:**
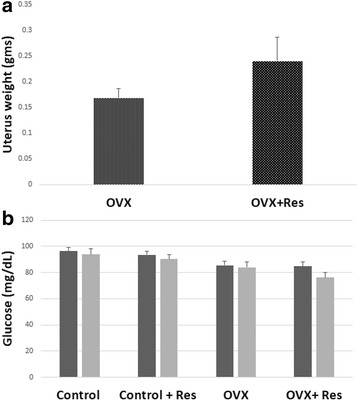
Effect of resveratrol on (**a**) uterus weight in OVX and OVX+ Res, (**b**) Glucose levels in Control and Control + Res, OVX and OVX + Res rats

Increase in the uterus weight was evident in the OVX + Res group in comparison to the OVX although the difference failed to attain any significance (*p >*0.05) (Fig. 3a).

###  Effect of resveratrol on biochemical parameters

**Fig. 4:**
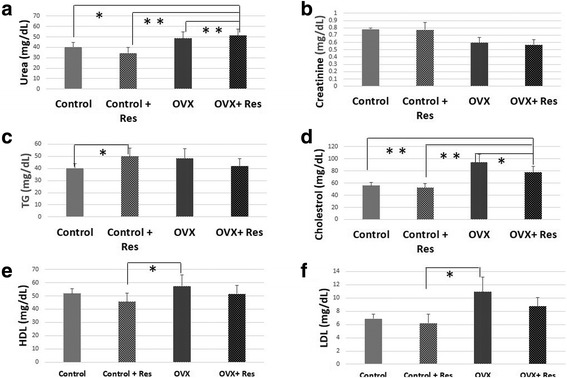
Effect of resveratrol on (**a**) Urea, (**b**) Creatinine, (**c**) Triglycerides (TG), (**d**) Cholestrol, (**e**) High density lipoprotein (HDL), (**f**) Low density lipoprotein (LDL) in control, control + Res, OVX and OVX + Res rats

Blood glucose levels of control, ovariectomized groups and their respective resveratrol treated groups were estimated at necropsy and we found a slight decrease in the glucose levels of the OVX rats treated with resveratrol though the change was not significant (*p >* 0.05) while no change was found in the control rats treated with resveratrol Fig. 3b. Resveratrol treatment brought about a slight decrease in the urea levels of the control rats though the change was not significant (*p >* 0.05) Fig. 4a. While the mean serum creatinine was within the normal range in all the animals it was lower in all the experimental groups compared to the control and resveratrol treated control group. Resveratrol treatment led to decrease in the creatinine levels in the control rats though the change was not significant (*p >* 0.05) Fig. 4b. A significant increase in the mean TG levels was observed in the control + RES rats in comparison to control rats (*p <* 0.05). Resveratrol brought about a small decrease in the TG levels of the OVX (*p >* 0.05) group though the change was not significant (*p >* 0.05) (Fig. 4c. Significant increase in the cholesterol levels was observed in the OVX rats (*p <* 0.001) in comparison to the control rats Fig. 4d. Resveratrol treatment brought about a decrease in the cholesterol levels of the control and OVX rats though the change was not significant (*p <* 0.05). A slight decrease was observed in the HDL levels of the control and OVX rats upon resveratrol treatment though the change was not significant (*p >* 0.05) Fig. 4e. A slight decrease in the LDL levels was observed in the control and OVX rats upon resveratrol treatment though the change was not significant (*p >* 0.05) Fig. 4f.

## Discussion

Much of the research on resveratrol has been carried out in animal models revealing its health benefits in cardiovascular risk factors, cancer and much more. It is already known for its anti - diabetic and anti- oxidant effects. Relatively little work has been done on its effect on body weight especially on the control rats. Menopause is accompanied by loss of estrogen which promotes a number of disorders including an increase in intra-abdominal fat [6]. In this study, OVX rats had significantly increased body weight in comparison to the control group. There are diverse targets such as SIRT1, AMPK, and cyclic adenosine monophosphate which acts through resveratrol and helps in increased oxidative phosphorylation, fatty acid oxidation, reduction in fatty acid synthesis and stimulate lipolysis [7]. This could be the explanation of the lower body weight observed in control + RES and OVX + RES rats. Recently it was found that increased energy expenditure in female mice could be attributed through SIRT1 which helps in transformation of white adipose tissue to the brown type [5]. Studies have also investigated the antiobesogenic effect of resveratrol by inhibition of *de novo* lipogenesis and adipose tissue fatty acid uptake mediated by lipoprotein lipase which plays a role in the reduction of the body fat [8, 9]. Resveratrol treatment was given for one month period in all the groups just before necropsy. In the OVX resveratrol treated group oral feeding with resveratrol was started after forty five days of OVX and in the case of control group the treatment was also started after forty five days. The rats were fed soya free diet to eliminate other soya derived phytoestrogens so that the effects seen are only due to the supplementation of the diet with resveratrol. This study used resveratrol for studying its therapeutic and not preventive potential especially in OVX rats unlike other studies available in the literature in which treatment of animals with resveratrol was initiated either before or concomitant with ovariectomization. We could not find much statistically significant values in the biochemical parameters because of the shorter duration of treatment and we did not start the treatment concomitant with ovariectomization. In this study, OVX rats exhibited a slight decrease in blood glucose levels in contrast to the control rats. There was a significant decrease in the blood glucose levels of the OVX+ RES group in comparison to the control group (*p <* 0.05). A drop in the blood glucose levels by resveratrol is accredited to the increased expression of GLUT4 leading to more intracellular glucose transport, and boosted glycogen synthase activity, resulting in greater liver glycogen accumulation [10]. We also saw that there was a minor reduction in the blood glucose levels though it was maintained near normal levels. There was a significant increase in the urea and cholesterol levels of the OVX compared with the control and control + RES rats, though they were in the normal range. Resveratrol had the potential to bring the down the cholesterol levels as resveratrol activates SIRT 1 which helps in inhibiting the uptake of intestinal cholesterol and promote deacetylation of liver receptor leading to reverse cholesterol transport [11]. Chen et al. incubated 3 T3-L1 pre-adipocytes for 48 h in varying concentrations (10, 20, 40 and 80 μM) of resveratrol they observed reduced lipid accumulation in a dose -dependent manner in these cells with the exception with the lowest dose of 10 μM [12]. Recently it was reported for the same cells that after 24 h incubation with resveratrol at concentrations of 25 and 50 μM, adipogenesis was inhibited [13]. There was an increase in the LDL levels of the OVX rats in contrast to the control rats. Resveratrol could bring down the LDL levels in the OVX treated group. This explains the decrease in serum cholesterol observed in OVX + RES rats. We also encountered a significant rise in triglycerides in the control + RES group which remains unexplained.

The results of this study clearly demonstrate that resveratrol supplementation in the presence of soya free diet helps in lowering blood glucose levels, urea, cholesterol, and LDL. An appreciable effect on the gain of body weight was seen upon resveratrol treatment in the control and OVX rats. It is likely that resveratrol do benefit the menopausal rats in reducing the gain in body weight but it is not good for healthy rats as we saw a reduction in the gain in body weight in the control rats. Uterus weight was measured because after ovariectomy the uterus is regressed. So we wanted to see whether resveratrol being a phytoestrogen had the potential to up bring the uterus weight.

Keeping in mind that resveratrol has multiple targets its consumption should be within recommended limits and should not be over enthusiastically consumed thinking of all its beneficial effects. One of the limitation of the study is that some other parameters should have been included so further research is needed on the dosage of resveratrol to maximize these benefits.

## Conclusion

With lower estrogen levels, animals tend to eat more and be less physically active. There was decrease in the gain in body weight when the OVX rats were treated with resveratrol which shows that in the OVX rats resveratrol is helping in balancing the body weight which was gained due to estrogen deficiency. Resveratrol significantly reduced the gain of body weight in the control rats and in OVX rats showing its antiobesogenic effects.

## Abbreviations

FFA: Free fatty acids
HDL: High-density lipoprotein
LDL: Low-density lipoprotein
OVX: Ovariectomized
RES: Resveratrol
